# Grasping Variance in Word Norms: Individual Differences in Motor Imagery and Semantic Ratings

**DOI:** 10.5334/joc.418

**Published:** 2025-01-07

**Authors:** Emiko J. Muraki, Sydney Born, Penny M. Pexman

**Affiliations:** 1 Department of Psychology, University of Calgary, Calgary, Canada; 2 Department of Psychology, Western University, London, Canada

**Keywords:** Semantic variables, Motor imagery, Individual differences, Lexical semantic processing

## Abstract

Word norming datasets have become an important resource for psycholinguistic research, and they are based on the underlying assumption that individual differences are inconsequential to the measurement of semantic dimensions. In this pre-registered study we tested this assumption by examining whether individual differences in motor imagery are related to variance in semantic ratings. We collected graspability ratings (i.e., how easily a word’s referent can be grasped using one hand) for 350 words and also had each participant complete a series of motor imagery questionnaires. Using linear mixed effect models we tested whether measures of motor imagery ability (e.g., the Florida Praxis Imagery Questionnaire and the Test of Ability in Movement Imagery for Hands) and motor imagery vividness (e.g., the Vividness of Movement Imagery Questionnaire 2) could account for variance (raw and absolute difference scores) in graspability ratings. We observed a significant relationship between motor imagery vividness and absolute rating difference scores, wherein people with more vivid motor imagery provided ratings that were further from the mean word ratings. However there was no relationship between motor imagery and raw rating difference scores. The results suggest that there are measurable systematic differences in how participants make sensorimotor semantic ratings, which has implications for how sensorimotor semantic word norms are used for investigations of lexical semantic processing.

A common methodological approach in psycholinguistics is to quantify dimensions of word meaning using large-scale norming studies. Word norms are often derived by asking people to rate words on a given semantic dimension using a Likert scale and then calculating the mean rating for each word based on the ratings of several participants. These word norm datasets afford several research advantages (see Keuleers & Balota, 2015 and Winter, 2022 for reviews), however, they have also been subject to criticism. One particular concern regards the midscale disagreement problem (Paisios et al., 2023; Pollock, 2018), in which words with ratings at the midpoint of a rating scale often have high standard deviations. High standard deviations indicate that these words elicit ratings on opposing ends of the ratings scale, resulting in a midpoint mean rating that reflects disagreement rather than a true measure of the dimension. There are several possible sources of variance that contribute to disagreement amongst raters. Words in norming studies are typically presented without context, which may result in inconsistent ratings for polysemous words if participants rate different meanings (Muraki, Reggin, et al., 2023; Winter, 2022) and there may be differences in how participants interpret and apply rating instructions (see Pollock, 2018 for examples). There is also an emerging body of research that considers rating variance related to individual differences such as age and gender. Although the goal of most word norming studies is to estimate values that should apply to all people by averaging across individuals, there is diversity in human experience. It is therefore worth measuring how much of the variability in ratings can be explained by systematic individual differences. In the present study we examined an individual difference that may be particularly salient for sensorimotor semantic dimensions, by testing whether motor imagery ability is related to variability in semantic ratings.

The extant literature examining individual differences in semantic ratings has focused mainly on age and gender differences in ratings of imageability, valence, and arousal. Across languages, older adults consistently rate words as more imageable than do younger adults (Ballot et al., 2022; Gilet et al., 2012; Grandy et al., 2020; Grühn & Smith, 2008) and younger adults tend to use a greater range of the likert scale when providing imageability ratings (Ballot et al., 2022; Grandy et al., 2020; Grühn & Smith, 2008). The findings are somewhat more mixed regarding emotional dimensions. In some cases older adults provided ratings that indicate more positive emotions (Grandy et al., 2020; Grühn & Smith, 2008), although the opposite pattern, of older adults providing more negative or more arousing ratings, has also been observed (Gobin et al., 2017; Teismann et al., 2020; Warriner et al., 2013), and one study found that middle-aged adults rated words as more positive than both the older and younger participants (Gilet et al., 2012). Other studies have investigated gender differences in ratings and found more modest differences. Teismann et al. (2020) found that women rated negative words as more arousing than did men, while they rated neutral and positive words as less arousing than did men and Warriner et al. (2013) found that men provided consistently higher ratings of valence, dominance, and arousal compared to women. These studies suggest that systematic individual differences in semantic ratings can be observed for some semantic dimensions.

More recently, researchers have begun to examine individual differences in motor imagery ability and their relationship to lexical semantic processing. The purpose of this work has been to interrogate the mechanisms of sensorimotor simulation during language processing. Motor imagery ability has been associated with faster and more accurate processing of words associated with more sensorimotor information compared to words with less information (Cayol et al., 2020; Muraki, Dahm, et al., 2023; Muraki & Pexman, 2021). In particular, mental imagery ability for object-directed actions appears to be related to participants’ sensitivity to the body-object interaction effect (BOI), wherein words whose referents are easier to interact with are processed more quickly and accurately (Siakaluk et al., 2008). Individuals with better imagery for object-directed actions have shown a BOI effect, whereas individuals with poorer imagery show no BOI effect (Muraki, Dahm, et al., 2023; Muraki & Pexman, 2021). Other measures of motor imagery, such as vividness of motor imagery, do not appear to be related to sensorimotor effects in language processing (Muraki & Pexman, 2021; Pavan & Baggio, 2013).

In the present study we extended this line of inquiry to examine whether individual differences in motor imagery ability are related to how individuals *rate* words on sensorimotor dimensions, rather than how quickly participants *process the meaning of* words based on their sensorimotor dimensions. If motor imagery ability is related to semantic ratings in systematic ways, this would suggest that individual variability should be evaluated and considered in future psycholinguistic studies.

We tested the relationship between motor imagery ability and graspability ratings (how easily a word’s referent can be grasped using one hand; Amsel et al., 2012; Heard et al., 2019). We selected graspability as our semantic dimension of interest for two reasons. First, the previously observed relationships between motor imagery ability and BOI effects in semantic processing were restricted to motor imagery for object directed actions, largely involving the hands. Second, graspability has been identified as the main subdimension of BOI (Heard et al., 2019). We tested two potential relationships between motor imagery and graspability ratings. First, we tested whether individuals with better motor imagery ability would provide higher graspability ratings compared to the mean, indicating that they are more inclined to rate words as graspable, regardless of which word they are rating. We also tested whether individuals with better motor imagery ability would provide more extreme ratings (i.e., provide ratings closer to the ends of the scale) for words that are either high or low in graspability, indicating stronger assessments of graspability in either direction of the scale. To do so, we selected motor imagery measures that were focused on fine-motor hand movements with and without objects (i.e., the Test of Ability in Movement Imagery with Hands or TAMI-h; Donoff et al., 2018) and that are sensitive to individual differences in sensorimotor effects in language processing (i.e. the Florida Praxis Imagery Questionnaire or FPIQ; Ochipa et al., 1997). We also included the Vividness of Movement Imagery Questionnaire (VMIQ-2; Roberts et al., 2008) and the Edinburgh Handedness Inventory (Oldfield, 1971) to control for imagery vividness and handedness effects, respectively. Our study hypotheses, design, and analysis plan were preregistered prior to data collection and are available at: https://osf.io/vcg5r.

## Method

### Participants

We recruited 164 monolingual English speakers (72 women, *M* age = 39.33, *SD* = 11.69, *M* years of education = 14.48, *SD* = 9.38) through Prolific. Participants were compensated at a rate of £9.00/hour. After our data cleaning procedures (see Results), 34 participants were removed from the study, leaving a final sample size of 130 participants (59 women, *M* age = 39.46, *SD* = 11.26, *M* years of education = 14.35, *SD* = 10.29).

### Stimuli

For the ratings task we selected 350 words from the Heard et al. (2019) graspability ratings dataset. The words were selected to include the entire range of ratings from Heard et al. (2019), with a preference for those words with larger standard deviations and words that were known by at least 95% of participants in the Brysbaert et al. (2019) word prevalence study. An additional six practice words were chosen that spanned the range of possible graspability rating values and had relatively low standard deviations in the Heard et al. (2019) dataset.

### Motor Imagery Questionnaires

#### Test of Ability in Movement Imagery with Hands (TAMI-h)

The TAMI-h assesses motor imagery abilities for simple hand movements (Donoff et al., 2018). Participants are instructed to imagine completing a sequence of five hand movements and then to choose a picture from several options that best reflects how they imagined their final hand position (TAMI-Hand subscale), or alternatively the object that would be easiest to grasp in that hand position (TAMI-Object subscale).

#### Florida Praxis Imagery Questionnaire (FPIQ)

The FPIQ was designed as a measure of imagery abilities in those with apraxia or similar movement disorders (Ochipa et al., 1997). The questionnaire has four subscales, each containing twelve questions, with two possible response options per question. Part A (Kinesthetic) asks which of two joints would move more while executing a particular task. Part B (Position) asks about the relative positioning of various objects or body parts while executing an action. Part C (Action) asks about the direction of movement of a tool or body part during an action. Part D (Object) asks about the size and shape of various parts of a tool.

#### Vividness of Motor Imagery Questionnaire-2 (VMIQ-2)

The VMIQ-2 is a self-report measure of motor imagery vividness (Roberts et al., 2008). The questionnaire asks participants to imagine 12 movements from three different perspectives: External (watching someone else complete the movement), Internal (watching yourself complete the movement while looking through your own eyes), and Kinesthetic Imagery (feeling yourself complete the movement). Participants rate vividness on a scale ranging from 1 (“Perfectly clear and as vivid as normal vision or feel of movement”) to 5 (“No image at all, you only ‘know’ that you are thinking of the skill”).

#### Edinburgh Handedness Inventory (EHI)

The EHI is a brief questionnaire to quantify handedness (Oldfield, 1971). It consists of ten questions displaying everyday activities where one hand is used preferentially. Participants are then instructed to put a checkmark (or two depending on the strength of preference) under the hand (left, right, or both) they prefer for each activity. Handedness scores are then calculated as the quotient of the difference and sum respectively of each hand’s total checkmarks.

### Procedure

Participants completed the online study through Qualtrics. All participants completed the ratings first, followed by the four questionnaires in a randomized order, so that the ratings would not be influenced or primed by the questionnaires. For the graspability ratings, participants were initially presented with the rating scale and instructions for the task. They completed six practice ratings, before rating all 350 words. Word order was randomized for each participant, with 25 words presented per page, and the rating choices beneath each word. The participants were then presented with brief instructions for the motor imagery section, followed by the four motor imagery questionnaires. Each questionnaire had preliminary instructions, and then questions presented in a fixed order. Instructions for the rating task are provided in the supplemental materials online.

## Results

All data cleaning and analyses were run in R (R Core Team, 2024). Our analysis plans and analysis scripts were pre-registered (https://osf.io/6ts3c/). The ratings data were cleaned to remove invalid responses (e.g., inattentive participants or bot responses) using the methods described in Pexman et al. (2019). We used the following exclusion criteria: participants were removed if they completed less than 33% of the ratings within a dimension (no exclusions), provided the same rating for 12 or more words in a row (*n* = 28), made ratings on the control words that were correlated with existing ratings on that dimension at less than *r* = 0.20 (no exclusions) or made ratings on all words that correlated less than *r* = 0.10 with the mean ratings collected in this study (no exclusions). We excluded an additional two participants because they did not fully complete the motor imagery measures.

### Graspability Ratings

We collected graspability ratings for 350 words (*M* graspability rating = 3.86, *SD* = 2.00). We assessed the rating reliability by calculating intraclass correlation coefficients by word and by participant. The ICC1 estimates indicate that a larger amount of variance in ratings is associated with the word (*r* = 0.61) than the participant (*r* = 0.06). The ICC2 estimates indicate high reliability in ratings across instances of words (*r* = 1.00) and participant (*r* = 0.96).

### Motor Imagery Measures

**Figure 1:**
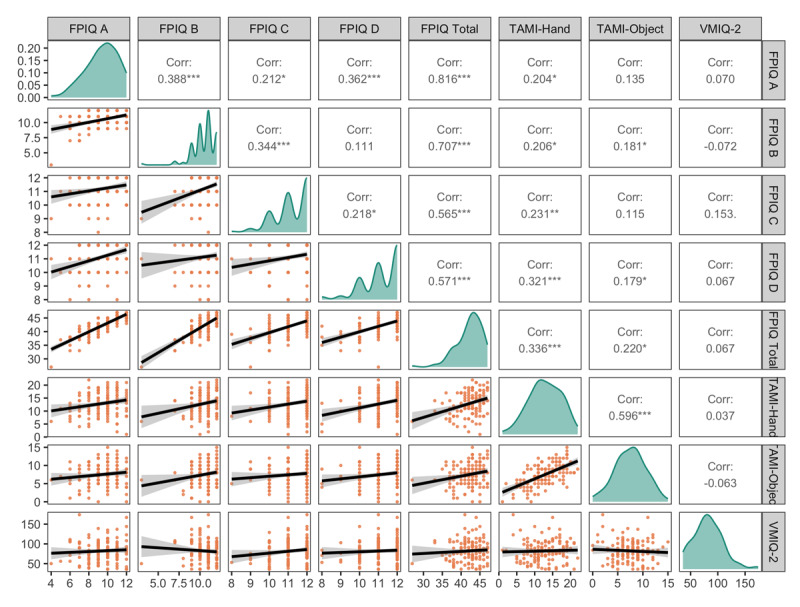
Distributions and Correlations of Motor Imagery Individual Difference Measures. *Note*. FPIQ = Florida Praxis Imagery Questionnaire; TAMI = Test of Ability in Movement Imagery; VMIQ-2 = Vividness of Movement Imagery Questionnaire 2.

The distributions of and correlations between the motor imagery measures are presented in Figure 1. Performance on the FPIQ (*M* score = 42.21, *SD* = 3.40) was negatively skewed indicating fairly high accuracy, which is typical for this measure in a healthy adult population (see Muraki, Dahm et al., 2023). The mean scores on each subscale were as follows: FPIQ A (Kinesthetic) *M* score = 9.41, *SD* = 1.72; FPIQ B (Position) *M* score = 10.47, *SD* = 1.32; FPIQ C (Action) *M* score = 11.19, *SD* = 0.89, and FPIQ D (Object) *M* score = 11.14, *SD* = 0.98. The scores on the TAMI-Hand (*M* score = 12.9, *SD* = 4.44) and TAMI-Object (*M* score = 7.5, *SD* = 3.06) were more normally distributed. The VMIQ-2 scores (*M* score = 82.18, *SD* = 26.73) were normally distributed with a slight positive skew. Low scores on the VMIQ-2 indicate more vivid motor imagery, whereas higher scores indicate less vivid imagery. The three objective measures of imagery ability (FPIQ, TAMI-Hand, TAMI-Object) were significantly correlated with one another, however the VMIQ-2 was not (see Figure 1).

#### Linear Mixed Effect Models

We examined whether the motor imagery measures accounted for variance in participant over/underestimates of ratings and absolute differences in participant ratings. We calculated participant over/underestimates of ratings by taking each participant’s rating for a word and subtracting the mean graspability based on all participant ratings. For this variable positive values indicate words for which the participant rated a word as more graspable than the mean and negative values indicate words for which participants rated a word as less graspable than the mean. We took the absolute value of over/underestimates of ratings as an index of the tendency to provide more extreme ratings compared to the mean.

Each dependent variable was entered into a linear mixed effects model that included the motor imagery measures and the participants’ handedness quotients as fixed effects. We included a random intercept for participant. We hypothesized that individuals with better motor imagery ability would provide more extreme ratings (i.e., provide ratings closer to the ends of the scale). Specifically, we predicted that motor imagery scores on the FPIQ or the FPIQ B sub-scale and the functionally-involved movement questions of the TAMI-Hand would be positively related to extremity of graspability ratings.

**Figure 2:**
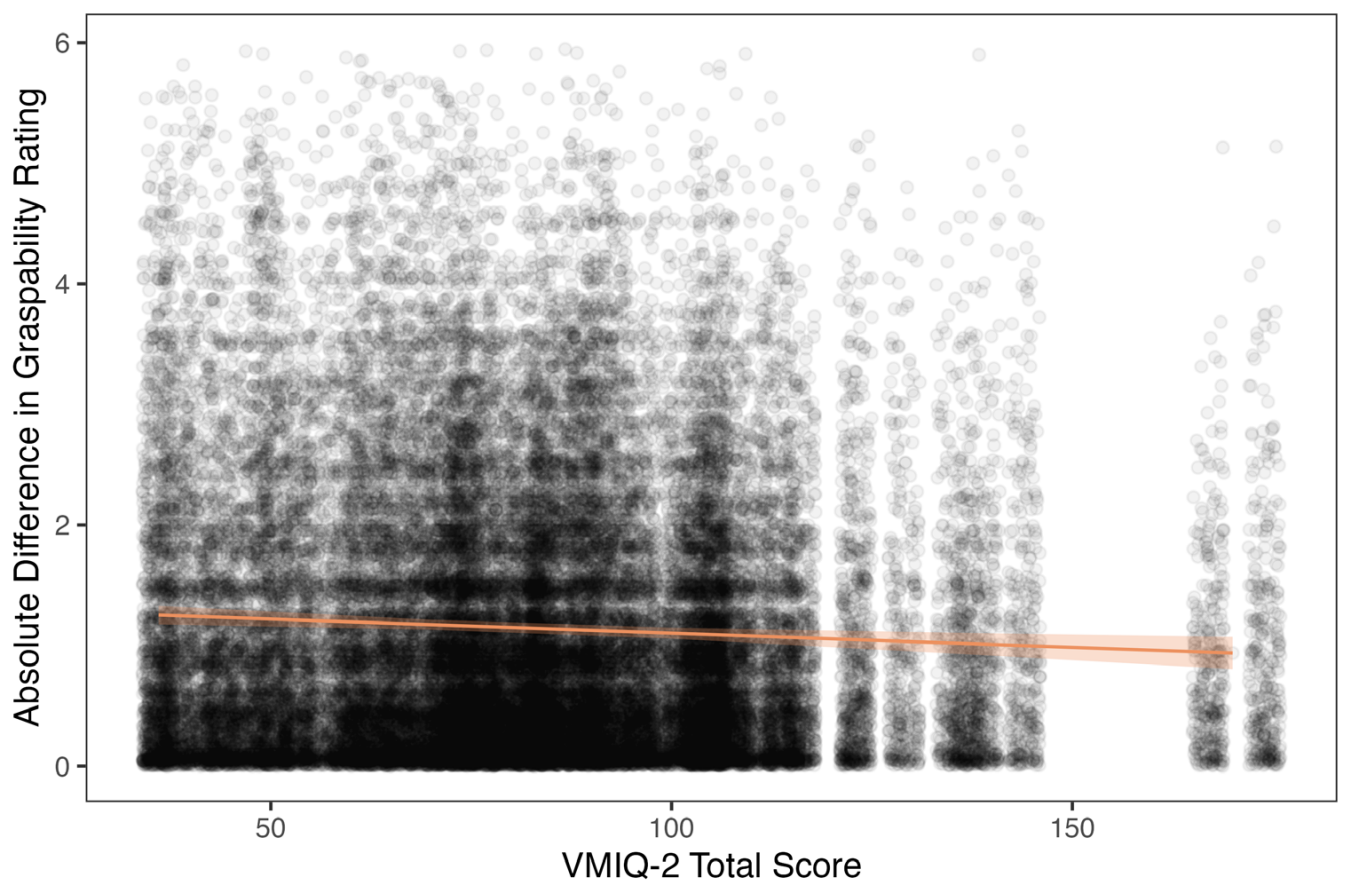
VMIQ-2 Relationship to Graspability Ratings Differences. *Note*. VMIQ-2 = Vividness of Movement Imagery Questionnaire 2. Points represent observed data, shading around the regression line represents the lower and upper 95% confidence limits. Lower VMIQ-2 scores indicate more vivid imagery.

The first model, predicting rating over/underestimates, accounted for 1.5% of the variance in graspability ratings (see Table 1). This increased to 17.6% variance when we include the random effects, suggesting that there is substantial individual variability that influenced ratings which was not captured by our fixed effects. None of the fixed effects were significant, but the fixed effect of VMIQ-2 score approached significance (*p* = .064). The second model, predicting absolute rating differences, accounted for 0.6% of variance in graspability ratings (see Table 1). This increased with the inclusion of random effects, but only to 4.2% of variance in absolute difference ratings, suggesting there is less individual variance in the absolute difference dependant variable. VMIQ-2 scores were a significant predictor^1^ of graspability rating, *b* = –0.002, *p* =.006, indicating that participants with more vivid movement imagery (i.e., lower VMIQ-2 scores) provided more extreme ratings (i.e., lower and higher than the mean; see Figure 2).

**Table 1:** Linear Mixed Effect Models Predicting Graspability Ratings.


	RATING (OVER/UNDERESTIMATE)			RATING (ABSOLUTE DIFFERENCE)		
	
PREDICTORS	B	CI	*P*	B	CI	*P*

(Intercept)	0.92	[–0.95, 2.79]	.335	1.59	[0.96, 2.22]	<.001

FPIQ A (Kinesthetic)	–0.05	[–0.13, 0.02]	.170	0.01	[–0.02, 0.03]	.582

FPIQ B (Position)	0.01	[–0.09, 0.11]	.854	–0.02	[–0.05, 0.02]	.354

FPIQ C (Action)	0.02	[–0.13, 0.16]	.818	0.01	[–0.04, 0.06]	.752

FPIQ D (Object)	–0.08	[–0.21, 0.05]	.232	–0.01	[–0.06, 0.03]	.611

TAMI – Hand	0.01	[–0.03, 0.04]	.749	–0.01	[–0.02, 0.00]	.074

TAMI – Object	–0.03	[–0.08, 0.01]	.147	0.01	[–0.01, 0.02]	.527

VMIQ-2	0.00	[–0.00, 0.01]	.064	–0.00	[–0.00, –0.00]	.002

Handedness Quotient	0.00	[–0.00, 0.00]	.778	–0.00	[–0.00, 0.00]	.302

**Random Effects**						

Residual	2.12			1.18		

Participant Intercept	0.41			0.04		

Observations	45460			45460		

Marginal *R*^2^/Conditional *R*^2^	0.015/0.176			0.006/0.042		


*Note*. CI = confidence interval; FPIQ = Florida Praxis Imagery Questionnaire; TAMI = Test of Ability in Movement Imagery; VMIQ-2 = Vividness of Movement Imagery Questionnaire 2. The marginal R2 includes only the variance from the fixed effects and the conditional R2 includes variance from both the fixed and random effects. The model equations were: Rating ~ FPIQ A + FPIQ B + FPIQ C + FPIQ D + TAMI-Hand + TAMI-Object + VMIQ-2 + Handedness Quotient + (1|Participant). *p*-values for fixed effects in the linear mixed effects model are calculated using Satterthwaite’s method. *N* participants = 130.

## Discussion

The purpose of this study was to test the relationship between motor imagery ability and graspability ratings, and in particular whether people with better motor imagery ability provide higher graspability ratings and/or more extreme graspability ratings. The second hypothesis was supported; absolute rating difference scores were significantly associated with vividness of motor imagery (VMIQ-2) and rating under/overestimates, which preserve the directionality of the rating, were not. However, although significant, individual differences in imagery vividness explained only a small amount of variance in graspability ratings (0.6%), and reliability estimates of the graspability ratings indicated that the graspability ratings were highly reliable, with very little variance associated with the participant.

The VMIQ-2 and the graspability ratings both used a Likert scale, meaning that the association we observed might simply reflect a tendency to use extreme ends of a response scale (Batchelor & Miao, 2016). We examined whether this was the case by testing whether absolute VMIQ-2 scores (calculated as the distance of each participant’s VMIQ-2 score from the mid-score reflecting only the middle of the Likert scale) explained variance in absolute difference ratings. Although absolute VMIQ-2 scores did explain significant variance in the ratings, it was not as effective a predictor as the VMIQ-2 score that includes both degree of scale extremity and the directionality of the self-report (i.e., vivid vs poor imagery). Thus, we conclude that, while the tendency to use extreme ends of the scale may influence this relationship, the relative vividness of imagery reported by participants does seem to matter. Individuals with more vivid imagery may be more confident in their assessments of whether a word’s referent is easily graspable, both when judging the referent to be not graspable and when judging the referent to be very graspable.

We did not observe an association between FPIQ scores and graspability ratings. In past research, this particular individual difference has been associated with BOI effects in semantic processing (Muraki, Dahm, et al., 2023; Muraki & Pexman, 2021). Instead, we observed an association between graspability ratings and self-reported imagery vividness, which has not previously been associated with response time or accuracy in semantic processing. This dissociation between motor imagery measures and their relationships to the different tasks suggests that the tasks differ in the *process* by which sensorimotor information is recruited. In the ratings task reported here, participants may need to recruit sensorimotor information in a conscious, voluntary manner to decide whether a word refers to something graspable, whereas in semantic processing tasks they may recruit this information in an unconscious, involuntary manner (see Muraki, Speed, et al., 2023 for further discussion). Imagery vividness, a subjective, conscious experience, may be more salient in, and thereby related to, tasks that involve conscious access to imagery.

The present findings suggest that individual differences may modestly affect the quantification and thereby subsequent use of semantic dimensions. There is evidence to suggest that systematic variability in semantic ratings has consequences for the nature of effects observed in lexical semantic processing. For example, semantic dimensions are better predictors of lexical semantic task performance when they have been rated by individuals of the same age as the task participants (e.g., task performance in older adults is better accounted for by ratings from older adults; Ballot et al., 2022; Balota et al., 2004) and semantic dimensions quantified from a child’s perspective are better predictors of word age of acquisition than ones quantified from an adult perspective (Muraki et al., 2022). Therefore, the differences in semantic ratings related to imagery vividness may be pertinent if researchers are interested in specific research questions associated with fine-grained semantic dimensions.

VMIQ-2 scores were a significant predictor of absolute differences in graspability ratings, suggesting that participants systematically differ in the sensorimotor semantic ratings they provide and that sensorimotor norms may include some noise that could be better explained for investigations of lexical semantic processing. There may be other variables which contribute to variability in semantic ratings, for instance age, gender, and personality characteristics (see Haro et al., 2024 for a recent investigation of these individual differences in lexical processing), that should be investigated to further reduce noise in word norming studies and attend to the midscale disagreement problem. Nonetheless, our findings suggest that individual differences in motor imagery only account for a small amount of variance in the ratings. While the results highlight the potential to better understand variability in human experience, they are also consistent with recent findings (Siew, 2024) that existing semantic rating norms can provide a reliable estimate of semantic dimensions for most investigations of lexical semantic processing.

## Data Accessibility Statement

Data generated or analyzed during this study are available in the OSF repository, https://osf.io/6ts3c/.

## Footnotes

This effect remained significant when age and gender were included in the model.

## References

[1] Amsel, B. D., Urbach, T. P., & Kutas, M. (2012). Perceptual and motor attribute ratings for 559 object concepts. Behavior Research Methods, 44(4), 1028–1041. 10.3758/s13428-012-0215-z 22729692 PMC3480996

[2] Ballot, C., Mathey, S., & Robert, C. (2022). Age-related evaluations of imageability and subjective frequency for 1286 neutral and emotional French words: Ratings by young, middle-aged, and older adults. Behavior Research Methods, 54(1), 196–215. 10.3758/s13428-021-01621-6 34131872

[3] Balota, D. A., Cortese, M. J., Sergent-Marshall, S. D., Spieler, D. H., & Yap, M. J. (2004). Visual word recognition of single-syllable words. Journal of Experimental Psychology-General, 133(2), 283–316. 10.1037/0096-3445.133.2.283 15149254

[4] Batchelor, J. H., & Miao, C. (2016). Extreme Response Style: A Meta-Analysis. Journal of Organizational Psychology, 16(2), Article 2. https://articlearchives.co/index.php/JOP/article/view/3628

[5] Brysbaert, M., Mandera, P., McCormick, S. F., & Keuleers, E. (2019). Word prevalence norms for 62,000 English lemmas. Behavior Research Methods, 51(2), 467–479. 10.3758/s13428-018-1077-9 29967979

[6] Cayol, Z., Rotival, C., Paulignan, Y., & Nazir, T. A. (2020). “Embodied” language processing: Mental motor imagery aptitude predicts word-definition skill for high but not for low imageable words in adolescents. Brain and Cognition, 145, 105628. 10.1016/j.bandc.2020.105628 33007685

[7] Donoff, C. M., Madan, C. R., & Singhal, A. (2018). Handedness effects of imagined fine motor movements. Laterality: Asymmetries of Body, Brain and Cognition, 23(2), 228–248. 10.1080/1357650X.2017.1354870 28724337

[8] Gilet, A.-L., Grühn, D., Studer, J., & Labouvie-Vief, G. (2012). Valence, arousal, and imagery ratings for 835 French attributes by young, middle-aged, and older adults: The French Emotional Evaluation List (FEEL). European Review of Applied Psychology, 62(3), 173–181. 10.1016/j.erap.2012.03.003

[9] Gobin, P., Camblats, A.-M., Faurous, W., & Mathey, S. (2017). Une base de l’émotionalité (valence, arousal, catégories) de 1286 mots français selon l’âge (EMA). European Review of Applied Psychology, 67(1), 25–42. 10.1016/j.erap.2016.12.001

[10] Grandy, T. H., Lindenberger, U., & Schmiedek, F. (2020). Vampires and nurses are rated differently by younger and older adults—Age-comparative norms of imageability and emotionality for about 2500 German nouns. Behavior Research Methods, 52(3), 980–989. 10.3758/s13428-019-01294-2 32052352 PMC7280351

[11] Grühn, D., & Smith, J. (2008). Characteristics for 200 words rated by young and older adults: Age-dependent evaluations of German adjectives (AGE). Behavior Research Methods, 40(4), 1088–1097. 10.3758/BRM.40.4.1088 19001400

[12] Haro, J., Hinojosa, J. A., & Ferré, P. (2024). The role of individual differences in emotional word recognition: Insights from a large-scale lexical decision study. Behavior Research Methods. 10.3758/s13428-024-02488-z PMC11525433 39231911

[13] Heard, A., Madan, C. R., Protzner, A. B., & Pexman, P. M. (2019). Getting a grip on sensorimotor effects in lexical-semantic processing. Behavior Research Methods, 51(1), 1–13. 10.3758/s13428-018-1072-1 29967978

[14] Keuleers, E., & Balota, D. A. (2015). Megastudies, crowdsourcing, and large datasets in psycholinguistics: An overview of recent developments. Quarterly Journal of Experimental Psychology, 68(8), 1457–1468. 10.1080/17470218.2015.1051065 25975773

[15] Muraki, E. J., Dahm, S. F., & Pexman, P. M. (2023). Meaning in hand: Investigating shared mechanisms of motor imagery and sensorimotor simulation in language processing. Cognition, 240, 105589. 10.1016/j.cognition.2023.105589 37566931

[16] Muraki, E. J., & Pexman, P. M. (2021). Simulating semantics: Are individual differences in motor imagery related to sensorimotor effects in language processing? Journal of Experimental Psychology: Learning, Memory, and Cognition, 47(12), 1939–1957. 10.1037/xlm0001039 34197170

[17] Muraki, E. J., Reggin, L. D., Feddema, C. Y., & Pexman, P. M. (2023). The Development of Abstract Word Meanings. Journal of Child Language, 1–13. 10.1017/S0305000923000569 37789718

[18] Muraki, E. J., Siddiqui, I. A., & Pexman, P. M. (2022). Quantifying children’s sensorimotor experience: Child body–object interaction ratings for 3359 English words. Behavior Research Methods, 54, 2864–2877. 10.3758/s13428-022-01798-4 35112287

[19] Muraki, E. J., Speed, L. J., & Pexman, P. M. (2023). Insights into embodied cognition and mental imagery from aphantasia. Nature Reviews Psychology, 1–15. 10.1038/s44159-023-00221-9

[20] Ochipa, C., Rapcsak, S. Z., Maher, L. M., Gonzalez Rothi, L. J., Bowers, D., & Heilman, K. M. (1997). Selective deficit of praxis imagery in ideomotor apraxia. Neurology, 49(2), 474–480. 10.1212/wnl.49.2.474 9270580

[21] Oldfield, R. C. (1971). The assessment and analysis of handedness: The Edinburgh Inventory. Neuropsychologia, 9, 97–113. 10.1016/0028-3932(71)90067-4 5146491

[22] Paisios, D., Huet, N., & Labeye, E. (2023). Addressing the Elephant in the Middle: Implications of the Midscale Disagreement Problem Through the Lens of Body-Object Interaction Ratings. Collabra: Psychology, 9(1), 84564. 10.1525/collabra.84564

[23] Pavan, A., & Baggio, G. (2013). Linguistic representations of motion do not depend on the visual motion system. Psychological Science, 24(2), 181–188. 10.1177/0956797612450882 23300229

[24] Pexman, P. M., Muraki, E., Sidhu, D. M., Siakaluk, P. D., & Yap, M. J. (2019). Quantifying sensorimotor experience: Body-object interaction ratings for more than 9,000 English words. Behavior Research Methods, 51(2), 453–466. 10.3758/s13428-018-1171-z 30484218

[25] Pollock, L. (2018). Statistical and methodological problems with concreteness and other semantic variables: A list memory experiment case study. Behavior Research Methods, 50(3), 1198–1216. 10.3758/s13428-017-0938-y 28707214 PMC5990559

[26] R Core Team. (2024). R: A language and environment for statistical computing. (Version 4.4.1) [Computer software]. R Foundation for Statistical Computing. https://www.R-project.org/

[27] Roberts, R., Callow, N., Hardy, L., Markland, D., & Bringer, J. (2008). Movement imagery ability: Development and assessment of a revised version of the Vividness of Movement Imagery Questionnaire. Journal of Sports & Exercise Psychology, 30, 200–221. https://doi.org/10/gf5crw; 10.1123/jsep.30.2.200 18490791

[28] Siakaluk, P. D., Pexman, P. M., Aguilera, L., Owen, W. J., & Sears, C. R. (2008). Evidence for the activation of sensorimotor information during visual word recognition: The body-object interaction effect. Cognition, 106(1), 433–443. 10.1016/j.cognition.2006.12.011 17258186

[29] Siew, C. S. Q. (2024). A comparison of word humor ratings across speakers of North American, British, and Singapore English. Memory & Cognition. 10.3758/s13421-024-01587-8 38865076

[30] Teismann, H., Kissler, J., & Berger, K. (2020). Investigating the roles of age, sex, depression, and anxiety for valence and arousal ratings of words: A population-based study. BMC Psychology, 8(1), 118. 10.1186/s40359-020-00485-3 33160414 PMC7648958

[31] Warriner, A. B., Kuperman, V., & Brysbaert, M. (2013). Norms of valence, arousal, and dominance for 13,915 English lemmas. Behavior Research Methods, 45(4), 1191–1207. 10.3758/s13428-012-0314-x 23404613

[32] Winter, B. (2022). Managing Semantic Norms for Cognitive Linguistics, Corpus Linguistics, and Lexicon Studies. In A. L. Berez-Kroeker, B. McDonnell, E. Koller, & L. B. Collister (Eds.), The Open Handbook of Linguistic Data Management (pp. 489–498). The MIT Press. 10.7551/mitpress/12200.003.0047

